# Composite biomaterial repair strategy to restore biomechanical function and reduce herniation risk in an *ex vivo* large animal model of intervertebral disc herniation with varying injury severity

**DOI:** 10.1371/journal.pone.0217357

**Published:** 2019-05-28

**Authors:** Warren W. Hom, Melanie Tschopp, Huizi A. Lin, Philip Nasser, Damien M. Laudier, Andrew C. Hecht, Steven B. Nicoll, James C. Iatridis

**Affiliations:** 1 Leni & Peter W. May Department of Orthopaedics, Icahn School of Medicine at Mount Sinai, New York, New York, United States of America; 2 Department of Biomedical Engineering, The City College of New York, New York, New York, United States of America; University of Pennsylvania, UNITED STATES

## Abstract

Back pain commonly arises from intervertebral disc (IVD) damage including annulus fibrosus (AF) defects and nucleus pulposus (NP) loss. Poor IVD healing motivates developing tissue engineering repair strategies. This study evaluated a composite injectable IVD biomaterial repair strategy using carboxymethylcellulose-methylcellulose (CMC-MC) and genipin-crosslinked fibrin (FibGen) that mimic NP and AF properties, respectively. Bovine *ex vivo* caudal IVDs were evaluated in cyclic compression-tension, torsion, and compression-to-failure tests to determine IVD biomechanical properties, height loss, and herniation risk following experimentally-induced severe herniation injury and discectomy (4 mm biopsy defect with 20% NP removed). FibGen with and without CMC-MC had failure strength similar to discectomy injury suggesting no increased risk compared to surgical procedures, yet no biomaterials improved axial or torsional biomechanical properties suggesting they were incapable of adequately restoring AF tension. FibGen had the largest failure strength and was further evaluated in additional discectomy injury models with varying AF defect types (2 mm biopsy, 4 mm cruciate, 4 mm biopsy) and NP removal volume (0%, 20%). All simulated discectomy defects significantly compromised failure strength and biomechanical properties. The 0% NP removal group had mean values of axial biomechanical properties closer to intact levels than defects with 20% NP removed but they were not statistically different and 0% NP removal also decreased failure strength. FibGen with and without CMC-MC failed at super-physiological stress levels above simulated discectomy suggesting repair with these tissue engineered biomaterials may perform better than discectomy alone, although restored biomechanical function may require additional healing with the potential application of these biomaterials as sealants and cell/drug delivery carriers.

## Introduction

Intervertebral disc (IVD) injuries can result in herniation when the central nucleus pulposus (NP) tissue protrudes or extrudes through defects in the surrounding annulus fibrosus (AF). Herniated IVD tissue can impinge upon surrounding nerves to cause radiculopathy with pain and disability in the back, neck, arms, and/or legs depending on the IVD level of the injury [1]. Conservative methods to address these symptoms include physical therapy and pain medication, but if these treatments fail then the next step is to remove the herniated tissue through discectomy surgery. There are approximately 300,000 annual discectomy procedures performed in the United States and this procedure is very successful for improving acute pain and disability due to neuropathy [2,3]. Current discectomy procedures do not replace the removed NP tissue or repair AF defects. Furthermore, the IVD is avascular and has a low cell-density, which contributes to its poor healing potential [4,5]. As such, there is a need to develop improved IVD repair strategies to prevent disc height loss, altered biomechanics, and accelerated degeneration from IVD injury and complications from discectomy procedures, including reherniation and recurrent pain at the same level [6–10].

Several structural biomaterials and hydrogels have been developed to repair or replace IVD tissues that are damaged or degenerated during injury, herniation, and degeneration but few have undergone rigorous biomechanical testing to assess functional restoration and reherniation risk [11,12]. The NP and AF each have distinct material properties and biomechanical roles and both must be addressed to fully repair the IVD. The NP has large swelling potential and exerts large intradiscal pressures [13–15] that put the fibrocartilaginous AF into circumferential tension which acts to constrain the NP. To achieve these distinct mechanical functions, the AF and NP have large differences in material properties and structure [8,16–18]. A successful IVD repair strategy similarly must achieve multiple mechanical functions to mimic the IVD and would therefore ideally incorporate two biomaterials: one to replace the removed NP and another to seal the AF and prevent reherniation. Such combination repair strategies are still in the early stages of development and currently there is one study by Borem *et al*. that utilized decellularized tissues to create an AF patch and an NP replacement scaffold [19] which demonstrated improvement in axial biomechanical properties [20] and another study by Sloan *et al*. which demonstrated that a combined hydrogel repair had the best restoration of NP hydration [21].

Injectable hydrogels are ideal candidates for IVD repair because they can fill in irregularly shaped defects and are amenable to minimally invasive procedures. Two previously investigated hydrogels which demonstrated promising IVD biomechanical restoration and biocompatibility were redox-polymerized carboxymethylcellulose-methylcellulose (CMC-MC) for NP replacement and a genipin-crosslinked fibrin (FibGen) hydrogel for AF repair. CMC-MC has high swelling potential and has demonstrated its ability to restore disc height and biomechanical restoration following a discectomy injury in bovine IVDs *ex vivo* [22,23]. FibGen has been tuned to match AF shear mechanical properties and has been shown to seal AF defects and resist reherniation under cyclic loading at physiological levels [24–29]. Both CMC-MC and FibGen are capable of rapid *in situ* gelation which is important for clinical translation when considering ease of application without extending surgical procedure times.

Herniation of NP tissue can occur through various AF defect types ranging in size from small tears up to large fissures spanning 50% or more of the IVD height. *Ex vivo* studies have been conducted to determine the biomechanical effects of varying AF defect sizes and found that biomechanical changes generally increase with AF defect size and require a defect spanning 40% or more of the IVD height in order to induce significant biomechanical changes [30–32]. However, those studies mainly focused on modeling IVD injury and degeneration and examined AF defects alone without trying to model discectomy procedures by removing NP tissue, and without efforts at repair. Therefore, there is limited data on whether the size and severity of AF defects have an effect on IVD biomechanical behaviors following discectomy surgery and how this influences repair performance.

This 2 part *ex vivo* biomechanical study evaluated how different biomaterial strategies influenced the biomechanical performance and failure strength of IVD injuries of varying severity. Part 1 focused on effects of biomaterial repair strategy and investigated the combined use of CMC-MC and FibGen to restore IVD biomechanical properties and failure strength back to intact levels following discectomy with an experimentally-induced severe defect and discectomy injury involving a 4-mm biopsy AF defect with 20% NP removal. Histological assessments determined if hydrogels filled void space and adhered to IVD tissues. Part 2 focused on AF defect type and NP volume removed and investigated whether varying AF defect size, type, and NP removal volume in the experimentally-induced injury and discectomy scenarios affected biomechanical performance and failure strength with FibGen, which was found to have lowest herniation risk from Part 1.

## Materials and methods

### Motion segment preparation and potting

Bovine caudal IVD motion segments from coccygeal levels cc2/3 to cc4/5 were isolated and prepared as previously described [26]. Briefly, oxtails were obtained from a local abattoir (Green Village Packing, Green Village, NJ) and the vertebral processes and surrounding muscles and ligaments were removed. Caudal motion segments were isolated and potted in poly(methyl methacrylate) and submerged in phosphate-buffered saline (PBS) (Fisher Scientific, Hampton, NH) at 4°C overnight under free-swelling conditions without a preload to allow full hydration before mechanical testing.

### Hydrogel fabrication and injection

FibGen was prepared as previously described [24]. Briefly, 200 mg of bovine fibrinogen (Sigma-Aldrich, St. Louis, MO) was dissolved in 1140 μL of PBS and mixed with 40 U of thrombin (Sigma-Aldrich, St. Louis, MO) and 8 mg of genipin (FUJIFILM Wako Chemicals U.S.A. Corporation, Richmond, VA) using a 4:1 dual barrel syringe and a mixing tip (Pac-Dent, Brea, CA). The tip of the syringe was then placed in the defect and slowly retracted as FibGen was injected to fill the void created by injury.

CMC and MC polymers (Sigma-Aldrich, St. Louis, MO) were methacrylated as previously described [23,33] and mixed along with 20 mM each of the redox initiators ammonium persulfate (Sigma-Aldrich) and N,N,N’,N’-tetramethylethylenediamine (Fisher Scientific, Hampton, NH) using a 1:1 dual barrel syringe (Pac-Dent, Brea, CA). The filled syringe was then warmed in a 37°C water bath for 30 minutes before injection into an injured motion segment. For the combined repair (Combo) samples, CMC-MC hydrogel was injected first and then the remaining void of the AF defect was filled in with FibGen. The volume of hydrogel injected per repair sample was carefully controlled to completely fill the defect to the AF periphery, as might be expected in a surgical repair setting. However, the precise volume injected was not measured and the amount may have varied depending on the size of the AF defect and the NP void space.

### Study design, mechanical testing protocol, and injury & repair procedures

**Fig 1:**
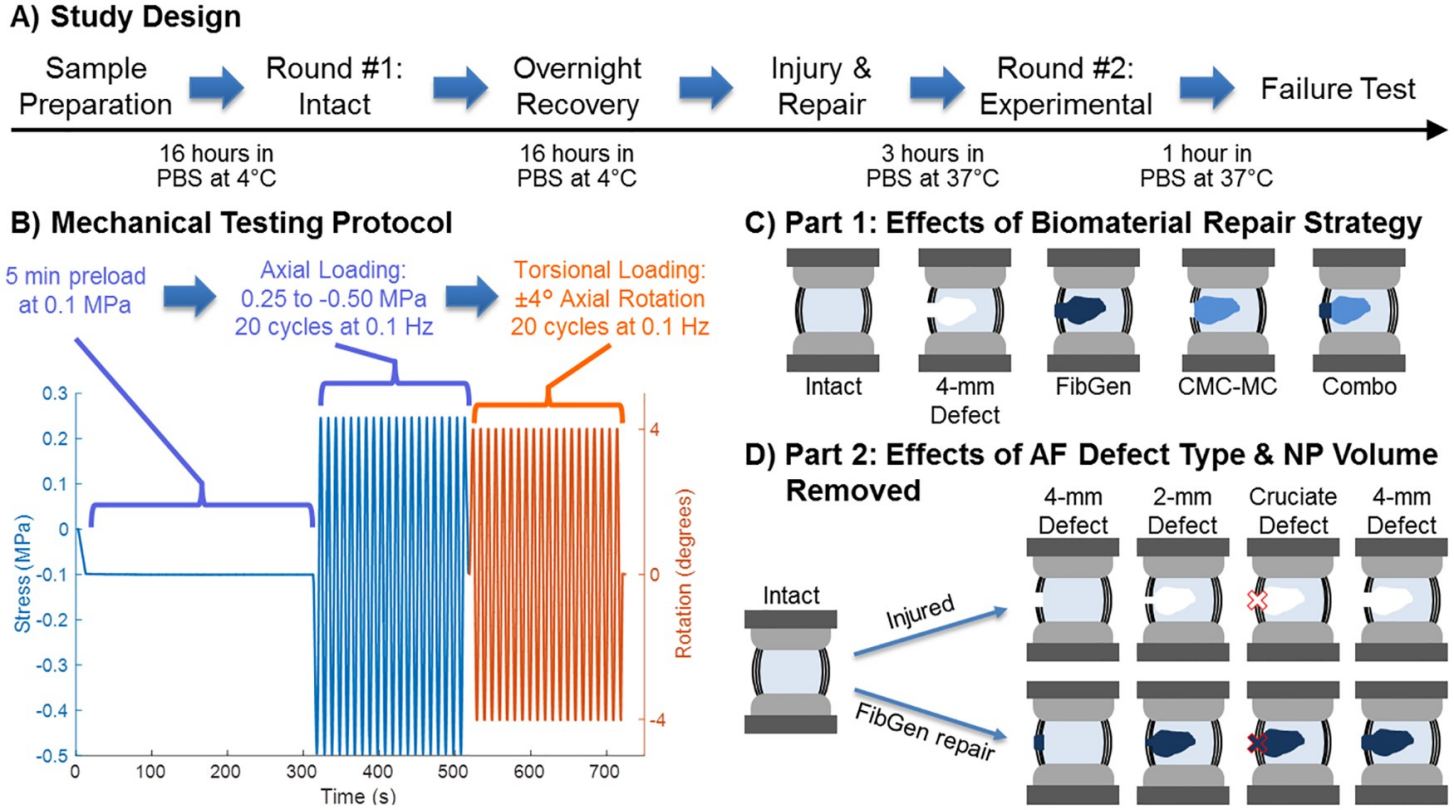
Schematic of study design, experimental groups, and loading protocol. The sequence of events for biomechanical testing (A) along with the loading protocol used for the two rounds of cyclic mechanical testing (B). Part 1 of this study tested bovine IVD motion segments divided into 5 groups (C) while Part 2 tested 8 groups (D).

For both Parts 1 and 2, all samples (n = 8–10 per group) were tested twice, first in the intact state to establish the initial biomechanical properties and then again in one of the experimental states before concluding with a failure test which assessed reherniation risk (Fig 1A). The biomechanical properties calculated from the second round of mechanical testing for each of the groups were all normalized to the values calculated from the first round of mechanical testing when every sample was in the intact state. All groups started with n = 10, yet some samples were excluded because of fibrotic, degenerated NP or preexisting endplate damage identified during injury creation or on x-ray images. Mechanical testing was performed with the MTS Bionix Servohydraulic Test system (MTS, Eden Parairie, MN) using a loading protocol that consisted of a 5 minute preload at 0.1 MPa followed by 20 cycles at 0.1 Hz of axial loading from 0.25 MPa of tension to -0.50 MPa of compression. Immediately after axial loading, the samples were then tested in torsion at ±4° of rotation for 20 cycles at 0.1 Hz (Fig 1B). These loading conditions are similar to those applied in previous studies [26,30,34] and represent stress magnitudes known to occur during physiological loading on human spines [14,15,17]. The 5 minute preload applied was sufficient to stabilize the rate of disc height change prior to starting cyclic loading, although full equilibrium would require longer loading periods. The samples were then removed from the testing system and submerged in PBS at 4°C for overnight recovery. The next morning, the samples were warmed to 37°C for 1 hour before performing the injuries and repairs. All mechanical testing was performed at room temperature.

Part 1: Effects of Biomaterial Repair Strategy consisted of 5 groups: Intact (n = 9), 4-mm Defect (n = 9), FibGen (n = 10), CMC-MC (n = 10), and combined repair (Combo, n = 10) (Fig 1C). Samples assigned to the Intact group were not injured. A 4-mm biopsy punch defect (4-mm Defect) was used in Part 1 to create the AF defect for the injured and repaired samples. The punch created a complete AF defect that involved a depth spanning through the whole AF thickness. After creating the AF defect, the healthy NP tissue was disrupted with a curette and then ~20% of the total NP was removed with a rongeur, based on weight. The amount of NP volume removed varied with IVD size. A preliminary test removed the NP from 15 intact IVDs and correlated the weight of the NP vs. the IVD diameter. Since there was no way of measuring the exact amount of NP in an intact disc prior to performing the injury, we used this correlation and removed between 0.15–0.20 g of NP depending on the IVD diameter, with an average removal of 0.188 ± 0.025 g.

Part 2: Effects of AF Defect Type and NP Removal Volume consisted of 7 groups: Intact (n = 9), 3 different AF defects (2-mm biopsy punch (n = 9), 4-mm biopsy punch (n = 9), and 4-mm cruciate (n = 10)), and the repair of each of the AF defects with FibGen (n = 10 per group) (Fig 1D). The cruciate defect (Cruciate Defect) was made with a #15 scalpel blade with each cut approximately 4 mm long. Biopsy defects were created with punches of 2 mm (2-mm Defect) and 4 mm (4-mm Defect) diameter. All AF defects in both parts of the study were created on the left posterolateral side of the IVD. As with Part 1, after creating the complete AF defects, the healthy NP was disrupted and ~20% of NP tissue removed.

To determine the influence of NP removal on severely injured IVDs and the ability of FibGen to restore AF radial tension, motion segments with the NP disrupted using a curette but not removed (0% NP Removed) were divided into two groups, 4-mm Defect (n = 8) and FibGen repair (n = 8), and tested using the previous mechanical loading protocol. Results from the 0% NP Removed groups were compared against the 4-mm Defect and FibGen samples which had 20% NP removed.

Following the injury, the samples were repaired using injection of the appropriate biomaterial or combination biomaterial, as described above. The combination repair included 2 separate syringes first filling the NP space with CMC-MC and next filling the AF defect space with FibGen. Following repairs, IVDs remained at room temperature for 10 minutes to allow initial gelation before they were submerged in PBS and kept at 37°C for 3 hours to allow full solidification of both hydrogels. All samples were then tested a second time following the same loading protocol used for the first test.

After the second round of testing, samples were subjected to failure testing which consisted of an axial compression to failure at a rate of 2 mm/min at 5° of bending with the left posterolateral side facing the open side of the wedge (to push NP towards he defect), using procedures as previously described [35].

### Biomechanical parameters

**Fig 2:**
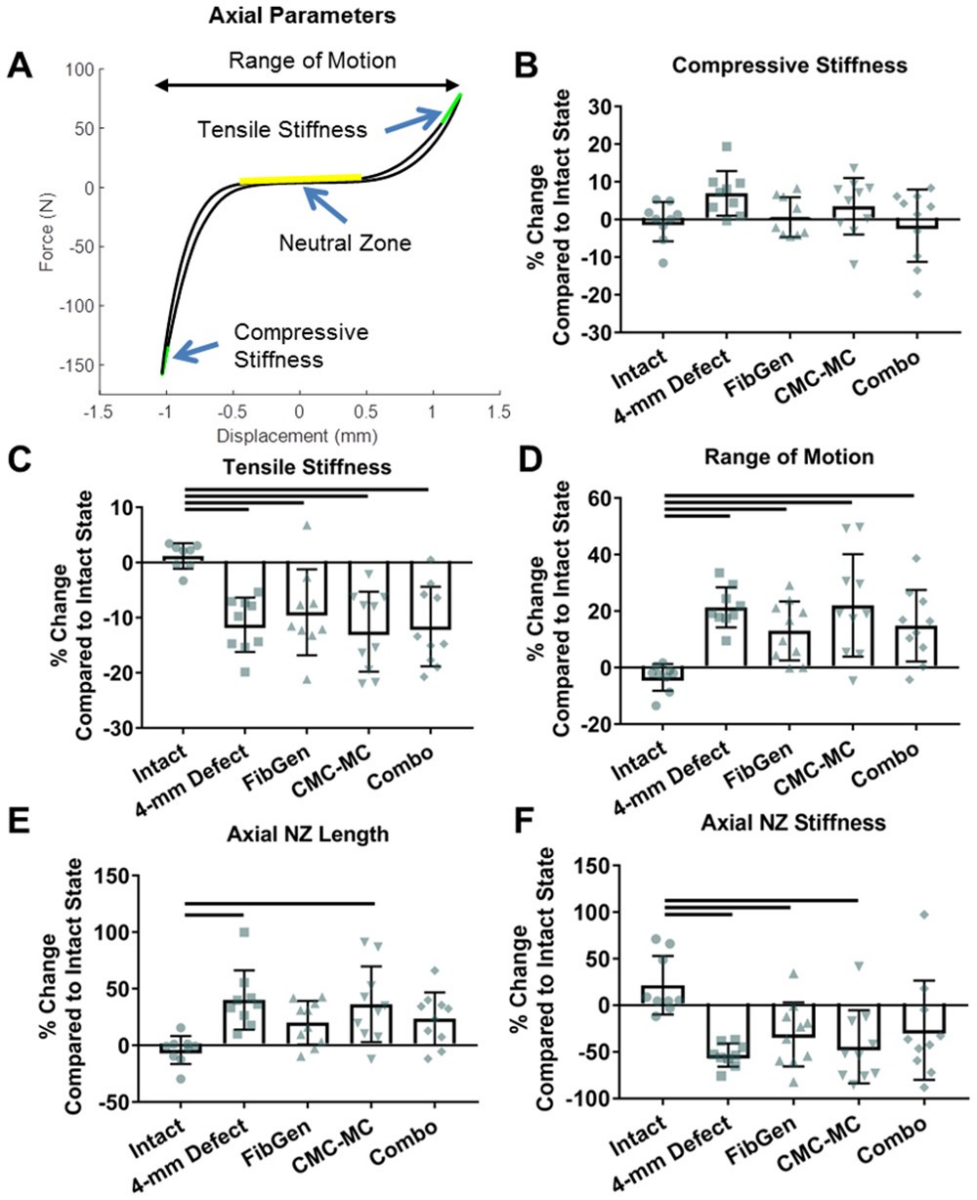
Part 1 axial biomechanical results. Representative force-displacement curve (A) which shows the regions of the curve analyzed to calculate range of motion (B), compressive stiffness (C), tensile stiffness (D), neutral zone length (E), and neutral zone stiffness (F). Bar indicates p < 0.05.

A custom MATLAB (Mathworks, Natrick, MA, USA) code was used to analyze axial and torsional data as follows. The last axial loading cycle was identified and the corresponding force-displacement curve was drawn (Fig 2A). Compressive and tensile stiffness values were defined as the slope of the linear regions of the force-displacement curve and calculated from the top 20% of the data points for the loading portion of the compressive and tensile curves. Range of motion (ROM) was defined as the total distance traveled from maximum compression to maximum tension in the last axial loading cycle.

Axial neutral zone (NZ) region was defined as the region of the force-displacement curve with the smallest slope. This region was found using the MATLAB code with a 15-point moving slope calculation along the compressive and tensile curves to find the position where a linear regression could be drawn with minimal slope. Once found, the NZ region was expanded by adding data points to each side of the initial 15 points until any additional data points would increase the standard error by 10%. This set of parameters for the algorithm were selected since they provided the NZ that best matched the NZ manually determined by three blinded observers. The axial displacement and slope of the NZ region were defined as the axial NZ length and NZ stiffness, respectively.

**Fig 3:**
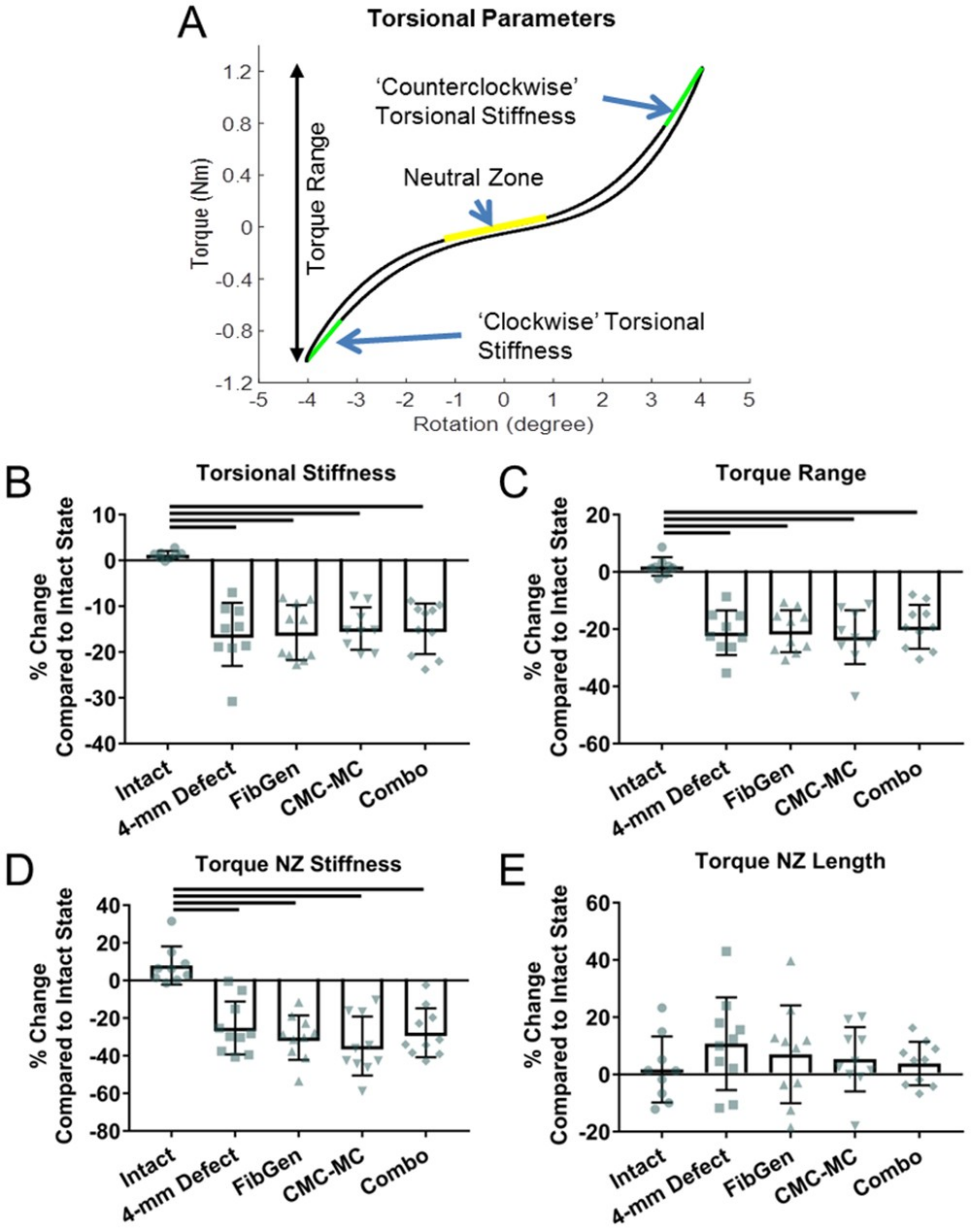
Part 1 torsional biomechanical results. Representative torque-rotation curve (A) which shows the regions of the curve analyzed to calculate torsional stiffness (B), torque range (C), torque neutral zone length (D), and torque neutral zone stiffness (E). Bar indicates p < 0.05.

For torsional properties, a torque-rotation curve (Fig 3A) for each sample was calculated from the last loading cycle. Torsional stiffness values for the clockwise and counterclockwise rotation directions were calculated with the same method used to calculate compressive and tensile stiffness but applied to the torque-rotation curve. Torsional stiffness values were calculated as the average of the clockwise and counterclockwise stiffness values since an equal degree of rotation was applied in each direction and the stiffness values from both directions within each sample were not statistically different (p = 0.125–0.776, paired t-tests). Torque range was defined as the total torque developed from +4° to -4° of rotation in the last cycle. Torsional NZ length and stiffness were calculated using the same method and similar code as for the axial NZ calculations.

### Disc height and failure strength analysis

Sagittal x-ray images (Faxitron Bioptics, LLC, Tucson, AZ) of each sample were taken before and after each round of cyclic mechanical testing. The convex curvature of bovine IVD endplates prevented straightforward measurement of the average disc height. Therefore, ImageJ was used to map the location of the superior and inferior endplates on the x-ray image and a custom MATLAB script was used to calculate the average distance between them. Disc height loss was defined as the percentage of IVD height loss experienced by the IVD after 20 cycles of axial tension-compression. This is a measure of effective engineering strain where the axial deformation measured at the end of the axial loading period of mechanical testing, which represented the change in disc height, was divided by the initial disc height of the IVD measured from the x-ray images.

Failure strength was defined as the effective stress at which the IVD failed. Two modes of failure were observed including NP herniation and, in the absence of herniation, endplate failure. The effective stress was calculated by dividing the force measured at the time of failure by the cross-sectional area of the IVD. Both modes of failure had characteristic peaks which were identified on stress-displacement curves, and herniation failures were further confirmed visually with video recordings.

### Histology

A subset of samples (n = 2–3) from Part 1 were selected for histological imaging following the second round of testing rather than undergoing failure testing. These samples were placed in zinc formalin (Z-Fix, Anatech, Battle Creek, MI) for 4 days before histological processing and embedding in methyl methacrylate [36]. Slides were stained with Gomori Hematoxylin, Alcian Blue, and Picrosirius Red and imaged with a Leica DVM6 stereomicroscope.

### Statistics

One-way ANOVA with Tukey’s post-hoc test was used in Part 1 to compare between groups. In Part 2 of the study, Two-way ANOVA with Bonferroni’s post-hoc was used to determine effects of the different AF defect types and FibGen repair status. Data presented as mean ± standard deviation along with individual data points. Statistical outliers were detected and removed using the integrated Robust regression and Outlier removal (ROUT) method [37] in GraphPad Prism 7 (GraphPad Software, San Diego, California) with Q = 1%. Inclusion or exclusion of the outliers did not affect the statistical results. Significance was set at p < 0.05 and all statistical analysis was performed using GraphPad Prism 7.

## Results

### Part 1 mechanical testing results

Compressive stiffness (Fig 2B) was not significantly altered by injury or repair (p>0.076) whereas tensile stiffness (Fig 2C) was significantly decreased due to injury (p<0.005) and not restored by any repair group (p>0.995). ROM (Fig 2D) for 4-mm Defect and all three repair groups were significantly increased compared to Intact values (p<0.032). Axial NZ length (Fig 2E) for 4-mm Defect and CMC-MC samples were increased (p<0.006) but FibGen and Combo repair samples were not significantly different from either Intact (p>0.110) or 4-mm Defect samples (p>0.378). Axial NZ stiffness (Fig 2F) was significantly decreased in 4-mm Defect, FibGen and CMC-MC samples (p<0.026).

Torsional stiffness, torque range, and torque NZ stiffness (Fig 3B–3D) were significantly decreased in 4-mm Defect and all repair groups (p<0.0001). Torque NZ length (Fig 3E) was not significantly affected by any group (p>0.581).

### Part 1 disc height and failure strength

**Fig 4:**
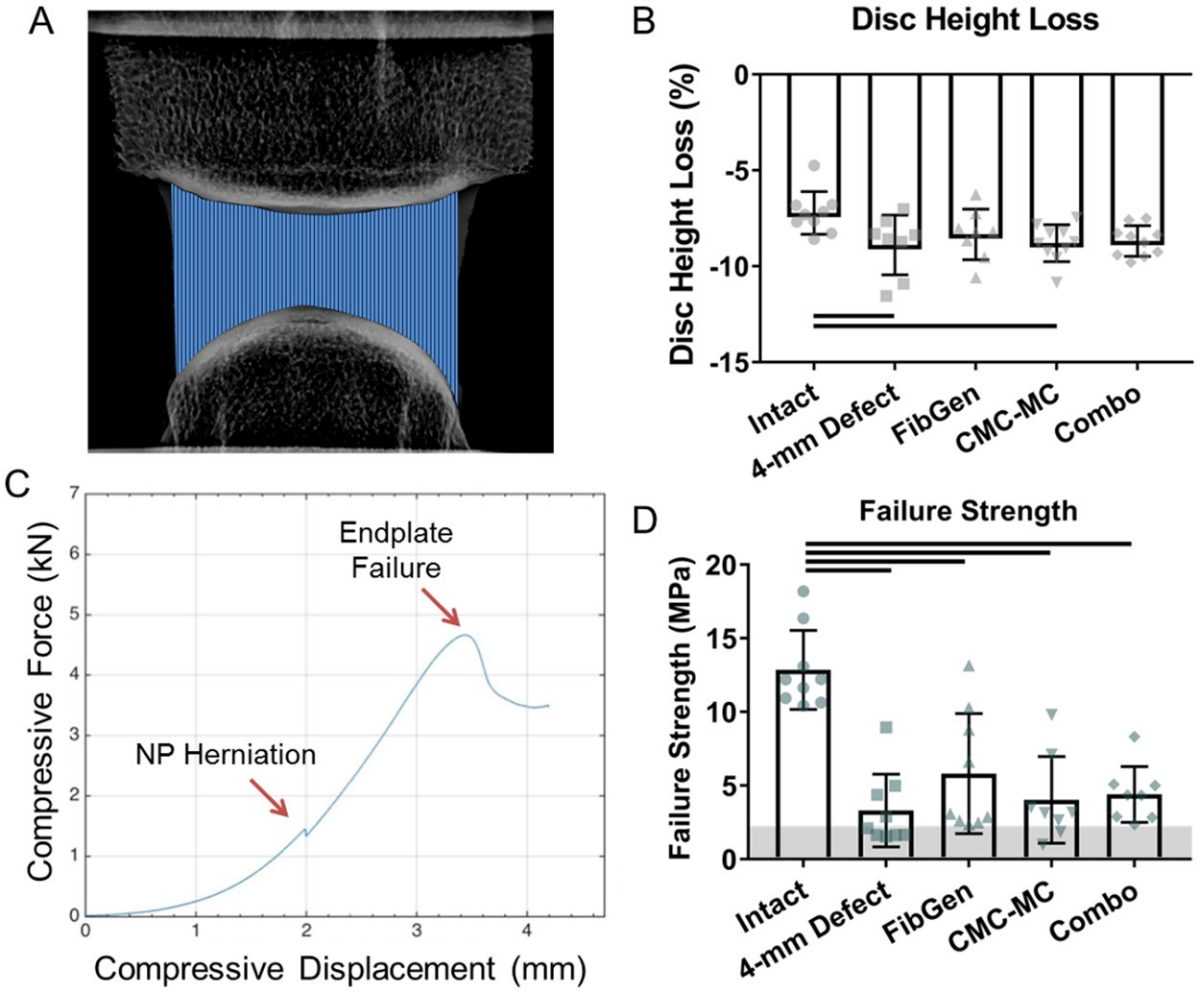
Disc height loss and failure strength for Part 1. X-ray images (A) were used to calculate disc height loss (B). Force-displacement curves (C) were used to calculate failure strength (D) of each sample. Horizontal grey shading indicates range of human physiological loads based on *in vivo* measurements of intradiscal pressure up to 2.3 MPa [14]. Bar indicates p < 0.05.

Disc height measurements obtained from x-ray images and MATLAB processing (Fig 4A) were used to calculate disc height loss (Fig 4B), which was significantly larger (i.e., disc height was smaller) for 4-mm Defect and CMC-MC samples (p<0.038). Disc height loss was not significantly different for FibGen and Combo samples as compared to either Intact (p>0.062) or the 4-mm Defect (p>0.872) samples. Prior to failure testing, visual assessment of the hydrogels did not reveal signs of cracking in FibGen or CMC-MC, although CMC-MC did appear to be closer to the edge of the AF defect (but still within the boundaries of the disc) than before mechanical testing.

In failure testing, samples failed either through herniation or endplate failure (Fig 4C). All 4-mm Defect and hydrogel repaired samples failed by herniation and had significantly reduced failure strength compared to Intact samples (p<0.0001) (Fig 4D), which all experienced endplate failure. None of the repaired samples had significant increased failure strength compared to 4-mm Defect samples (p>0.380), which represents the simulated discectomy condition. Failure strength was also larger than the physiological stress levels for IVDs.

### Morphology and histology

**Fig 5:**
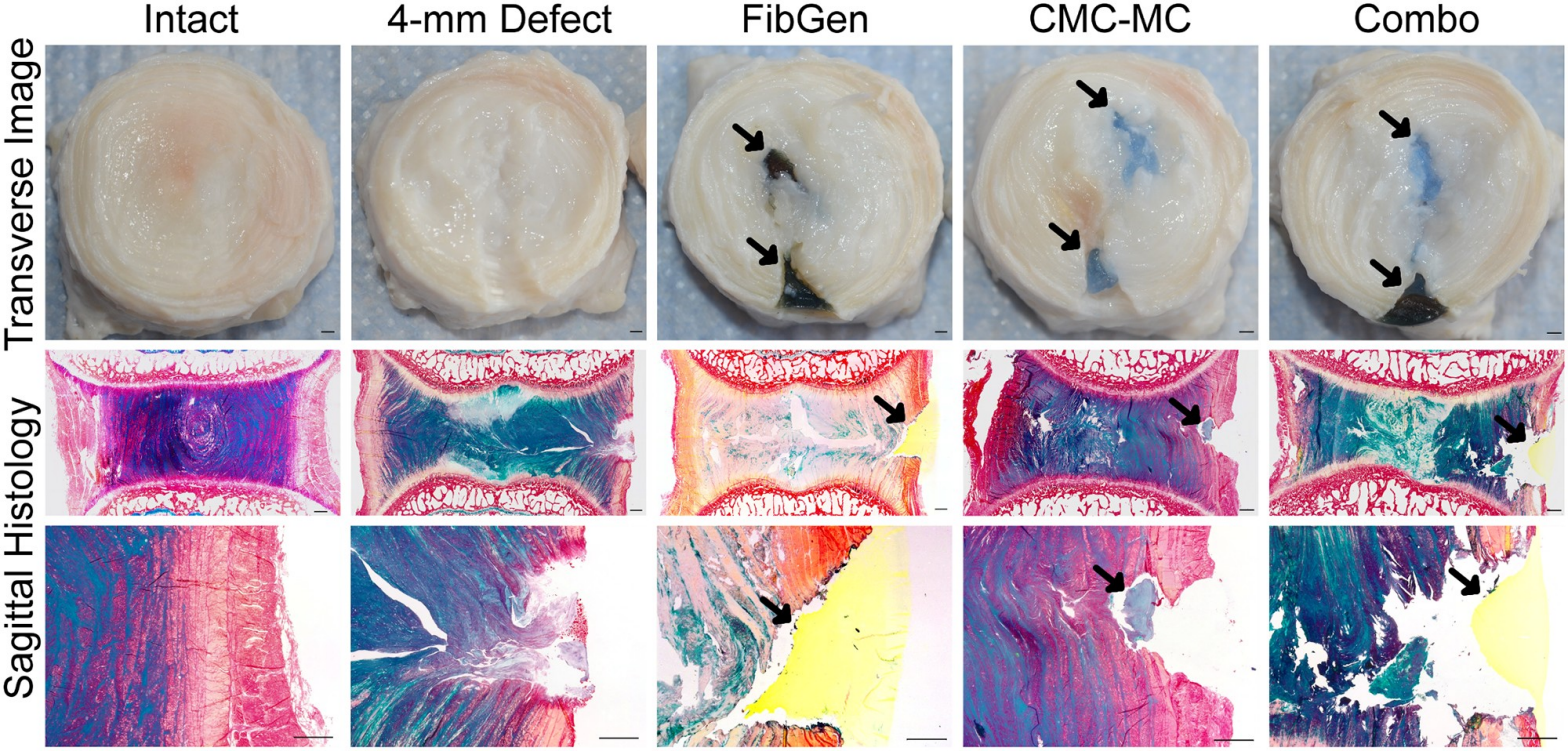
Qualitative assessment of hydrogel repair. Samples were cut transversely to determine extent of injury and repair. Sagittal histological sections were stained with picrosirius red and alcian blue to determine the amount of adhesion between native IVD tissue and the hydrogel repairs. Scale bar = 1 mm. Black arrows indicate hydrogel in situ.

In samples cut transversely prior to histological fixation (Fig 5), FibGen and CMC-MC can be seen within the IVD as dark and light blue hydrogels, respectively. Hydrogels and native IVD tissues swelled so there were no obvious void spaces. The stained images of sagittal sections show some void spaces following the fixation and staining processes which highlighted NP damage from the injury procedures. FibGen filled the AF defect space and remained adherent to the outer AF. CMC-MC was adjacent to native IVD tissues without obvious adhesion.

### Part 2 mechanical testing results

**Fig 6:**
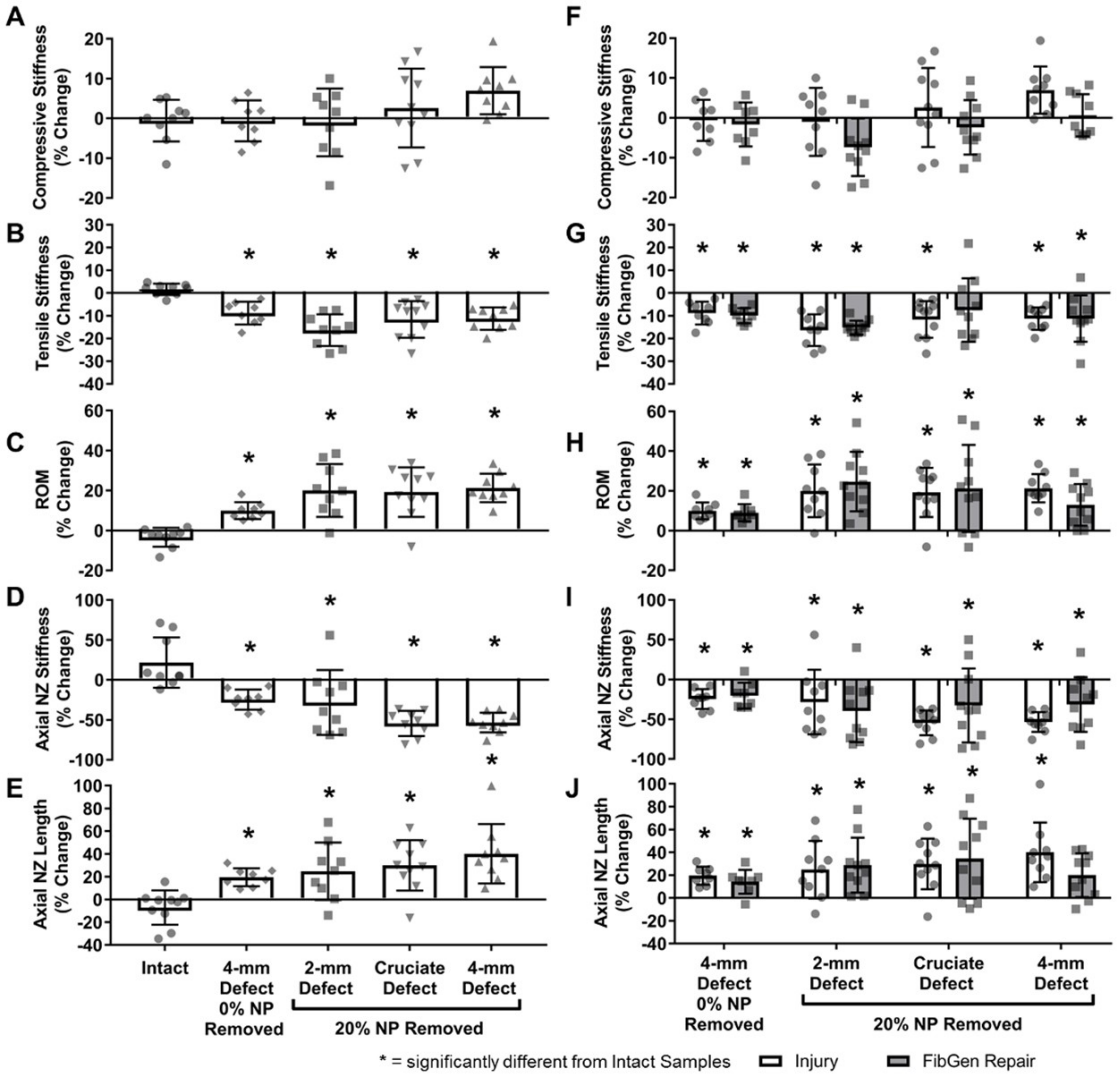
Part 2 axial biomechanical results. The compressive stiffness (A) was not significantly altered from intact levels by any of the three AF defects. Tensile stiffness (B), range of motion (C), axial neutral zone length (D), and axial neutral zone length (E) were significantly altered by each of the AF defects but there were no significant differences between the AF defects. There was no significant improvement with the addition of FibGen (F-J). * = significantly different from Intact Samples.

All axial biomechanical parameters measured were significantly altered with injury for all defect types assessed (p<0.040) (Fig 6A–6E), except for compressive stiffness which had no change (p>0.206). No significant differences between injury types were observed (p>0.083). FibGen repair of each injury type (Fig 6F–6J) had no significant improvements for most axial parameters (p>0.205) except for the tensile stiffness of the Cruciate FibGen repair and the axial NZ length of the 4-mm FibGen repair which both demonstrated partial restoration back to Intact levels since all injuries significantly changed biomechanical properties from Intact levels but FibGen did not (p>0.184).

**Fig 7:**
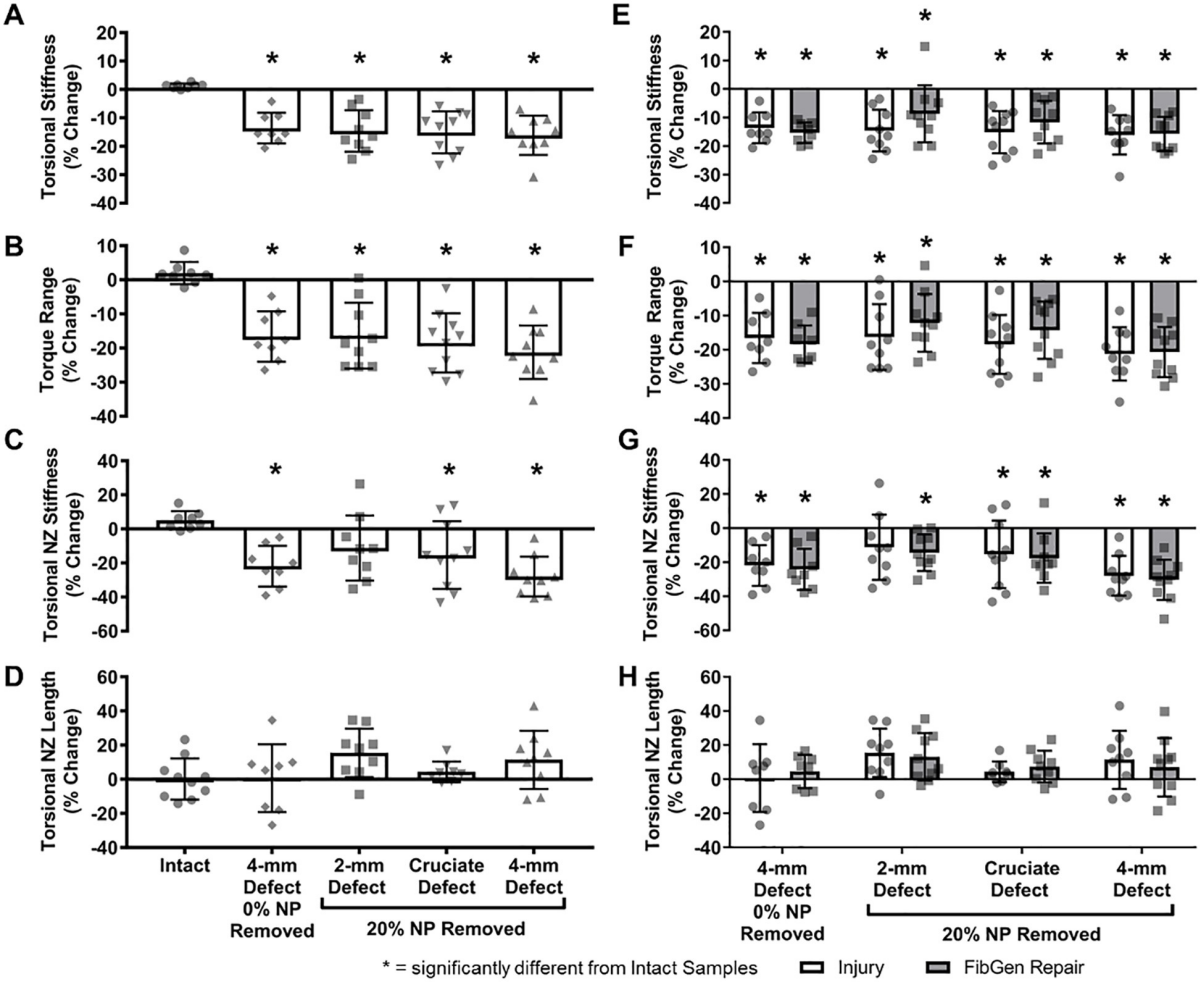
Part 2 torsional biomechanical results. The torque range (A), torsional stiffness (B), and torsional neutral zone (C) were significantly decreased from intact levels due to injury but there were no differences between groups. Torsional neutral zone length (D) was not significantly altered from intact levels by any group. FibGen repair of each defect type (E-H) did not provide any significant restoration. * = significantly different from Intact Samples.

Torsional stiffness and torque range decreased due to injury (p<0.0002), with no differences between the AF defects (p>0.669) (Fig 7A and 7B). Torsional NZ stiffness decreased for the 4-mm Defect (p = 0.0005) and Cruciate Defect (p = 0.049) but the 2-mm Defect was not significantly different from Intact values (p = 0.190) (Fig 7C). Torsional NZ length had no significant differences between Intact or injured samples (p>0.167) (Fig 7D). FibGen repairs of the injuries (Fig 7E–7H) showed no significant differences compared to injured samples (p>0.269). Volume of NP removal did not significantly affect axial or torsional biomechanics of the 4-mm Defect (p>0.109).

### Part 2 disc height and failure strength

**Fig 8:**
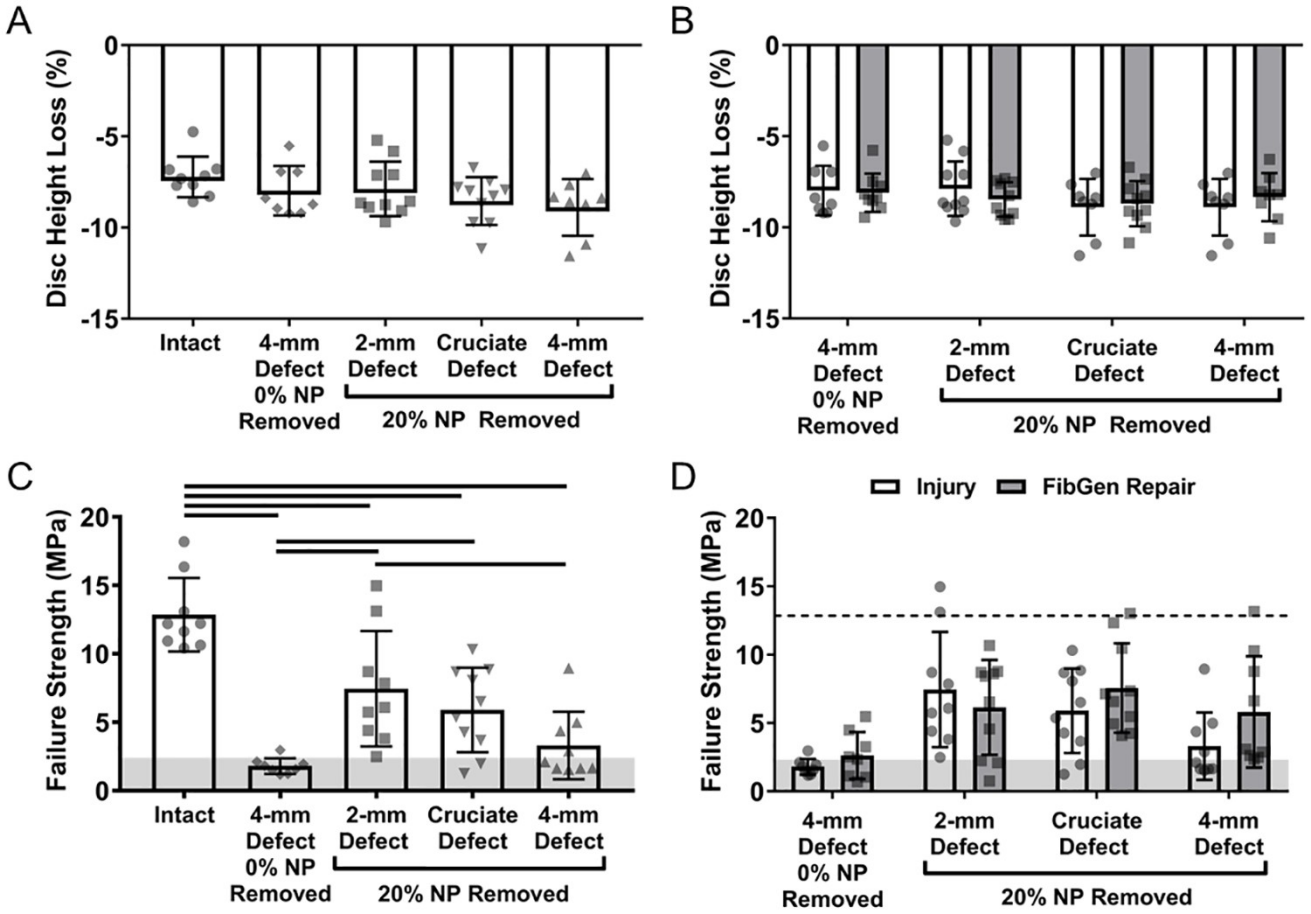
Part 2 disc height loss and failure strength. Disc height for injured (A) and FibGen repaired samples (B) were not significantly different from intact levels or different between groups. Failure strength was significantly decreased from intact by all four AF defects (C) but the 2 mm Defect had a greater failure strength than the 4 mm Defect at both 0% and 20% NP removed. The Cruciate Defect group also had greater failure strength than 4 mm Defect with 0% NP removed. Failure strength for FibGen repaired samples (D) were not significantly greater than the injured samples. Horizontal grey shading (C,D) indicates range of human physiological loads. Dashed line indicates mean of Intact values. Solid bars indicates p < 0.05.

No significant differences in disc height loss were observed between the Intact and injured groups (p>0.110) (Fig 8A), likely because of the free swelling recovery and high NP swelling propensity of these healthy bovine caudal IVDs. Since the injury groups had no significant differences, the disc height loss of the FibGen repairs of each defect type also had no significant differences between Intact and injured samples (p>0.180) (Fig 8B). Failure strength of the injured samples was significantly decreased from Intact levels (p<0.003) (Fig 8C). Among the three defect types that had 20% NP removed, the 2-mm Defect samples had significantly greater failure strength than the 4-mm Defect group (p = 0.032), highlighting that the 4-mm Defect was most severe. Furthermore, two out of the nine 2-mm Defect samples had endplate failure rather than herniation. All 4-mm and Cruciate Defect samples herniated. The 4-mm Defect with 0% NP removed all herniated and had significantly reduced failure strength compared to 2-mm Defect (p = 0.002) and Cruciate Defect samples (p = 0.037). FibGen repair did not significantly improve the failure strength for any of the injury types (p>0.360) but values of the FibGen 4-mm Defect and FibGen Cruciate groups were above the injury levels and were all above physiological levels of loading typically experienced *in vivo* (Fig 8D). FibGen repairs of 4-mm and Cruciate Defects all herniated while two out of the ten 2-mm FibGen repaired samples failed via endplate instead of herniation.

## Discussion

Discectomy procedures alleviate many symptoms associated with IVD herniation but do not address the damage and loss of NP and AF tissues which can result in reherniation and recurrent pain [6–10]. This 2 part biomechanical study developed and characterized injectable IVD biomaterial repair strategies capable of being applied during discectomy procedures to repair herniated IVDs. CMC-MC as an NP hydrogel replacement and FibGen as an AF sealant were applied. Part 1 evaluated effects of biomaterial repair strategy using hydrogel alone and in combination with large AF defects and NP tissue removal that simulate herniation and an ‘aggressive’ discectomy procedure. The combined hydrogel repair did not significantly improve IVD biomechanical properties suggesting no repair strategy was capable of restoring NP swelling and AF tension. However, failure strength of all repair strategies were greater than physiological loading levels (discussed below) and mean failure strength was largest for the FibGen group, although it was not significantly different from other groups and had the greatest variability. Part 2 evaluated effects of AF defect type and NP removal volume to determine their influence on biomechanical behaviors and FibGen repair capacity. Axial and torsional biomechanical properties were not significantly different between the three AF defects tested suggesting that amount of NP volume removed was more important than the AF defect type in determining the extent of biomechanical changes. Failure strength was greater for the 2-mm Defect and Cruciate Defect, highlighting that AF defect size and type was an important determinant of herniation potential. FibGen repair resulted in greater failure strength for larger defects indicating the importance of an AF sealant for a large defect.

AF and NP have distinct biomechanical properties and both of these tissues are disrupted and removed during herniation injury and discectomy procedures. The NP has high swelling capacity and exerts hydrostatic pressure, which places the surrounding AF fibers in circumferential tension [13,14]. When AF structural integrity is compromised, the NP pressurization is lost and IVD biomechanical properties are significantly altered [30,32,38]. Few studies investigate a combination repair and we hypothesized that a combination biomaterial repair strategy following simulated herniation and discectomy injury was necessary to restore NP hydrostatic pressure and AF tension and return biomechanical properties to the Intact condition. CMC-MC was selected as the NP replacement hydrogel due to its swelling potential [22] and FibGen was selected as the AF sealant for its ability to resist reherniation under physiological loads [26–29]. Both hydrogels were also previously determined to be biocompatible, injectable and capable of *in situ* gelation suggesting they show promise for application to augment current discectomy procedures [25,26,33,39]. The gross morphological images and sagittal histology performed in this study confirmed the ability of CMC-MC and FibGen to gel *in situ*. Imaging demonstrated that FibGen adhered to the native tissue while CMC-MC remained in place without adhering with the native tissue. A similar combination repair strategy was previously applied to rat coccygeal motion segments demonstrating that the NP replacement material had largest effects on biomechanical properties, highlighting the importance of NP swelling on axial biomechanical properties [21]. Biomaterial strategies involving composite repairs that mimic the whole IVD structure are also under development, although most of these strategies are being developed for a fusion alternative rather than a herniation repair [12,40,41].

Part 1 of this study applied a 4-mm biopsy punch injury spanning ~50% of the disc height and 20% NP removal, as might be observed with a severe herniation and aggressive discectomy. This severe defect altered nearly all biomechanical parameters with reduced tensile stiffness, torsional stiffness and torque range and increased axial range of motion. Multiple NZ properties were also affected for both axial and torsional tests. Interestingly, compressive stiffness was not significantly altered in the 4-mm Defect group or by the amount of NP removal. We believe the relatively small % difference (4-mm Defect group with 6.96±5.93% change) in compressive stiffness can be attributed to the healthy NP tissue undergoing free swelling to fill the void space and partially restore intradiscal pressure even with the large AF defect and removal of ~20% of the NP. The three hydrogel repair groups did not restore any axial or torsional parameter back to Intact levels and these groups were also not different from injury. The lack of restoration of torsional properties suggests that no biomaterial strategies were able to restore AF tension. The lack of restoration of axial biomechanical properties to Intact levels further suggests no biomaterial strategies were able to restore NP pressurization.

Prior studies, which used the same axial loading magnitudes and similar durations as this study, showed that repair of IVD defects with FibGen and CMC-MC significantly improved axial biomechanical properties, and that FibGen had a trend of improved torsional properties [23,26,27]. In this study, neither FibGen and CMC-MC nor the combination repair restored axial or torsional biomechanical properties. We believe these differences between studies are most likely attributed to differences in injury severity since this study involving larger AF defects and 20% NP removal (as compared to less NP removal in the study by Likhitpanichkul *et al*. [26] and less severe AF defects used by Varma *et al*. [23]). Results therefore highlight the importance of controlling for IVD injury severity in study design as well as a continuing need to develop repair strategies for more severe injuries.

Part 2 determined that different AF defect types had similar effects on biomechanical properties and that FibGen had little biomechanical improvement on any AF defects. AF defects from herniation observed during discectomy procedures vary in size and, in some cases, the AF defect is difficult to find and the surgeon has to create an incision to remove the protruding NP tissue [42]. Several biomechanical studies have also determined how different sizes and shapes of AF injury influence biomechanical changes in models of IVD degeneration [30–32,38,43], yet few evaluate how different injury models influence failure strength, which relates to IVD herniation risk [44,45]. The biopsy punch injuries selected for this study represent severe AF defects that can be seen clinically whereas the cruciate injury represents smaller AF tears or the incision surgeons make when the AF is still intact. The 4-mm and 2-mm biopsy punch defects were selected to induce injuries of 50% or 25% of the disc height, respectively. These percentages were selected based on a prior study by Elliot *et al*. which demonstrated that needle puncture injuries of 52% disc height caused greater biomechanical changes than injuries that were 26% of the disc height [30]. The Cruciate Defect involved 4-mm scalpel cuts that also spanned 50% of the disc height, cutting similar numbers of fibers as the 4-mm Defect but retaining the cut AF tissue. FibGen was selected to repair the AF defects in Part 2 because it had the most promising biomechanical restoration and was easier to apply than the Combo repair, which had similar performance as FibGen. All 3 defect models of Part 2 significantly diminished axial and torsional biomechanical parameters but were not significantly different from each other. This indicates that IVD biomechanical properties were equally altered regardless of the size or type of AF defect present after discectomy. Therefore, it is likely that the amount of NP removal dominates the influence of AF defect type on biomechanical properties, particularly since all AF defects were somewhat severe. This is contrary to previous studies which found that an AF defect size of 40% or more of the disc height is necessary to induce significant biomechanical changes [30]. However, those studies were modeling IVD degeneration and used needle puncture injuries with no NP removal whereas the injuries induced in this study did remove NP tissue to model the condition of the IVD after discectomy. We conclude that biomechanical changes in this study were driven largely by the amount of NP tissue removed once the AF was substantially disrupted, and that improved biomechanical performance following severe injuries requires restoration of both NP swelling and AF tension that our biomaterial strategies had little capacity to do in a severe injury model.

To determine whether FibGen could restore AF circumferential tension with NP tissue in place, IVDs with 0% NP removed were tested with the 4-mm Defect type and 4-mm FibGen repair conditions. Although the average change in axial and torsional properties for 0% NP removed samples was lower than injured samples with 20% NP removed, the four injury groups were not significantly different. FibGen repair of the 4-mm Defect with 0% NP removed did not provide significant biomechanical restoration. These results suggest that FibGen was unable to restore AF radial tension even when healthy NP tissue remained in place and was able to swell. Restoring AF tension might be possible if IVDs are unloaded prior to AF repair in order to allow restoration of AF pre-stress and/or NP pressurization with resumption of loading. However, such a strategy was not employed because it would be very difficult to apply in a clinical scenario.

Biomaterial repairs performed better for herniation risk and disc height loss than for biomechanical properties. Herniation risk and disc height loss are likely to be more important than biomechanical properties in improving clinical outcomes since IVD height loss and herniation risk are key factors that could directly impact nerve compression and must be addressed for clinical translation of any IVD repair strategy. Herniation risk was assessed by failure testing in which the IVD was compressed at 5° of posterolateral flexion until failure which was defined as NP herniation, hydrogel extrusion, or endplate fracture [35]. Although FibGen, CMC-MC and the Combo repair were unable to significantly improve failure strength compared to the injured condition, values for FibGen and Combo repairs were all above 2.3 MPa which is the upper range of physiological loading typically experienced by lumbar IVDs during moderate levels of physical activity [14]. On the other hand, half of the 4-mm Defect samples herniated within this range of physiological loading. This suggests that FibGen and the Combo repair may provide protection against reherniation even though the results were not statistically significant. CMC-MC is not an adhesive hydrogel and the 4-mm biopsy punch defect is very large and spanned 50% of the disc height so it was unsurprising that there was no improvement in failure strength highlighting that injectable CMC-MC is likely to be preferable for early IVD degeneration conditions with small IVD defects and little potential for herniation. Part 2 compared three AF defect types and found the 2-mm Defect and Cruciate Defect groups had greater failure strength than the 4-mm Defect, although the Cruciate Defect group was not significantly different from the 4-mm Defect. The 4-mm Defect was also used to assess the impact of the volume of NP removed on failure strength. Although the mean failure strength of the 0% NP removed group was lower than the 20% NP removed group, and most of the samples herniated at physiological loading levels, the two groups were not significantly different. This indicates that size and relative severity of the AF defect are important factors when considering the reherniation of any repair strategy and that the amount of NP removed in severe AF defects may also influence biomechanics and failure strength. Intact IVDs failed via endplate fracture rather than AF rupture and herniation, and this suggests it may never be possible to fully restore failure strength back to Intact levels using injectable hydrogels alone. In that case, the goal should be to prevent repairs from failing at physiological levels of loading while introducing bioactive factors or cells to promote healing.

Disc height loss is a common symptom of herniation and discectomy due to removal of NP tissue and this can lead to nerve root impingement and radicular pain. In clinical scenarios three months after discectomy, an 18% loss of disc height was observed which increased to 26% disc height loss two years after surgery [7]. In this study, the magnitude of disc height loss only reached 9% in injured samples and was not significantly different from Intact samples. A previous study has demonstrated that loading magnitude and duration both affect disc height loss [46]. As such, we believe that these disc height loss measurements are not so sensitive since the short duration of low-force loading and free-swelling recovery used in this study was not sufficient to simulate the disc height loss that would be observed under physiological conditions that more severely test these biomaterials. For more accurate IVD height measurements, we recommend application of a longer axial load such as during long-term organ culture experiments or creep experiments, since our prior studies showed both FibGen and CMC-MC were able to reduce or prevent disc height loss using those test protocols [23,26].

One of the limitations of this study is the relatively high variance observed. There are several sources of potential variance in this complex study of in situ repair using multiple biomaterials. We evaluated biomaterial formulations from different batches and found them to have relatively low variance. Previous studies by Amin *et al*. have attributed approximately 20% of the biomechanical variation to biological variability, experimental technique, and injury mechanisms [47,48]. Biological, anatomical, and technical factors in the multi-step potting and testing processes all contribute to variability and were controlled for in this study by normalizing each sample to their intact state and by having the same person, who was well-practiced, perform the same procedures to reduce this precision error. Relative injury severity also contributed to variance since NP removal volume was difficult to precisely control and since we used a fixed-size defect that did not account for variations in disc heights and diameters between samples. Differences in hydrogel repair procedures, such as the varied amounts of hydrogel injected, were an additional source of variation indicated by the repair groups having the largest variation for many variables.

Additional limitations may be related to the testing protocol. Axial and torsional loading were selected since they are physiologically relevant loading conditions and because torsional biomechanical properties are sensitive to AF integrity while axial biomechanical properties are sensitive to NP pressurization and AF integrity [8]. However, physiological loading of the IVD involves loading in six degrees of freedom and at different loading rates, which can affect biomechanical properties [49]. Although no significant differences between the different AF defect types were observed at a loading frequency of 0.1 Hz, testing with different loading frequencies or measuring viscoelastic parameters are interesting future investigations to further characterize injury effects and repair potential. The amount of IVD hydration can influence biomechanical properties [50] and the 5 minute preload applied prior to cyclic loading in this study may not have been sufficient to establish equilibrium in all samples. The addition of a recovery period between the axial and torsional tests in future studies would restore any potential hydration loss induced by the cyclic axial loading. While the defect models used in this study simulate clinically relevant AF defects, studies using human IVDs with facet joints intact which increase torsional stiffness and bear part of the compressive loads [51,52] would be more representative of *in vivo* biomechanical changes caused by injury and repair. Intact and healthy facet joints in the human spine may limit the magnitude of biomechanical changes caused by AF defects with the facets sharing part of the applied loads.

## Conclusion

This study investigated the combination of CMC-MC, an NP replacement, and FibGen, an AF sealant to restore IVD biomechanics after discectomy by restoring NP pressurization and AF tension. Although the biomechanical properties were not restored back to Intact levels, FibGen and the combined hydrogel remained adherent in the large 4-mm AF defect during axial and torsional cyclic loading and had failure strength above moderate levels of physiological loading. CMC-MC and FibGen have both previously demonstrated the ability to support the encapsulation of cells [28,33] and future work will continue to develop both hydrogels for sustained delivery of cells and soluble therapeutic factors with *in vivo* assessments. Results also demonstrated that NP removal volume had a greater impact on biomechanical dysfunction and that AF defect type had a larger impact on failure risk with implications for discectomy procedures with cruciate cuts exhibiting higher failure strength than similarly sized biopsy defects. IVD repair with FibGen showed the greatest increase in failure strength for nearly all conditions suggesting it is particularly important for large AF defects, although small defects had high failure strength and did not show an increase with repair. Variability observed in the results also highlights the need to optimize delivery methods as well as the biomaterial being delivered. No biomaterials were capable of recovering AF tension and NP swelling to restore biomechanical function, suggesting healing may be required to achieve this objective. The current study and literature lead us to conclude that FibGen and CMC-MC remain biomaterial candidates with potential benefit as sealants with low reherniation risk and reduced disc height loss. Therefore, their use as a carrier to deliver cells and bioactive factors that promote healing may be the most promising method to promote healing and allow for restored IVD biomechanical function.
